# Metagenomic next-generation sequencing in tuberculous periprosthetic joint infection: pathogen-detection yield and clinical characteristics compared with common bacterial PJI

**DOI:** 10.3389/fcimb.2026.1935713

**Published:** 2026-10-07

**Authors:** Jiayu Li, Yuntao Liao, Xiaolin Li, Lan Lin, Yafei Liu, Wenbo Li, Zida Huang, Zhaoyang Wu, Xinyu Fang, Wenming Zhang

**Affiliations:** 1 Department of Orthopaedic Surgery, The First Affiliated Hospital of Fujian Medical University, Fuzhou, China; 2 Department of Orthopaedic Surgery, National Regional Medical Center, Binhai Campus of the First Affiliated Hospital, Fujian Medical University, Fuzhou, China; 3 Fujian Provincial Institute of Orthopedics, The First Affiliated Hospital, Fujian Medical University, Fuzhou, China; 4 Fujian Orthopedic Bone and Joint Disease and Sports Rehabilitation Clinical Medical Research Center, The First Affiliated Hospital of Fujian Medical University, Fuzhou, China; 5 Department of Orthopaedic Surgery, Wuping County Hospital, Longyan, China

**Keywords:** Charlson comorbidity index, metagenomic next-generation sequencing, pathogen-detection yield, periprosthetic joint infection, synovial fluid polymorphonuclear neutrophil percentage, tuberculous periprosthetic joint infection

## Abstract

**Objective:**

To compare the pathogen-detection yield of metagenomic next-generation sequencing (mNGS) and conventional culture in tuberculous periprosthetic joint infection (TB-PJI) and common bacterial PJI (CB-PJI), and to characterize clinical differences and exploratory associations with TB-PJI.

**Methods:**

A retrospective cohort included 197 patients with periprosthetic joint infection treated between January 2016 and June 2024 who underwent both conventional culture and mNGS during the same diagnostic or surgical episode. Patients were classified as CB-PJI (n=178) or TB-PJI (n=19). Paired pathogen-detection rates were compared using McNemar’s test. Exploratory multivariable associations with TB-PJI were assessed using Firth penalized logistic regression with the Charlson Comorbidity Index (CCI) modeled continuously.

**Results:**

In CB-PJI, mNGS showed a higher pathogen-detection rate than conventional bacterial culture (94.4% vs. 78.1%, P<0.001). In TB-PJI, Mycobacterium tuberculosis complex (MTBC) was detected by mNGS in 17/19 cases (89.5%) and recovered by mycobacterial culture in 5/19 cases (26.3%; P<0.001); 12/19 cases were mNGS-positive/mycobacterial-culture-negative. In the primary exploratory Firth model, each 1-point increase in CCI was associated with higher odds of TB-PJI (aOR=2.39, 95% CI: 1.51-4.10, P<0.001), whereas each 10% increase in SF PMN% was associated with lower odds (aOR=0.722, 95% CI: 0.564-0.917, P = 0.009); BMI was not statistically significant. Drug-related complications were more frequent in TB-PJI (42.1% vs. 11.8%, P = 0.002), while the 2-year infection recurrence rate did not differ significantly (15.8% vs. 7.9%, P = 0.216).

**Conclusion:**

In this selected retrospective cohort, mNGS provided a higher pathogen-detection yield than conventional culture, particularly for TB-PJI. Higher CCI and lower SF PMN% were exploratory clinical associations with TB-PJI and should not be interpreted as validated diagnostic thresholds. mNGS should be considered an adjunct to mycobacterial culture, molecular testing, and histopathology rather than a stand-alone diagnostic test.

## 1 Introduction

Periprosthetic joint infection (PJI) represents one of the most devastating and challenging complications following total joint arthroplasty, contributing substantially to functional impairment and socioeconomic burden (Izakovicova et al., 2019; Aftab et al., 2025). Accurate and timely identification of the causative pathogen is critical for the therapeutic success of PJI management (Wang et al., 2024). Although the vast majority of PJIs are secondary to common bacterial pathogens such as Staphylococcus or Streptococcus species, a considerable proportion of clinical cases involve complex infections caused by atypical, fastidious, or cryptic microorganisms (Izakovicova et al., 2019; Fröschen et al., 2022).

Tuberculous periprosthetic joint infection (TB-PJI) is a rare but clinically challenging entity within the PJI spectrum (Lo et al., 2021; Auñon et al., 2024; Ortega-Portas et al., 2025; Nazli et al., 2026). Its insidious onset and nonspecific manifestations can delay etiological recognition (Bi et al., 2019; Auñon et al., 2024). Mycobacterial culture has limited pathogen-detection yield and requires prolonged incubation, while the low organism burden in osteoarticular specimens further complicates recovery of MTBC (Indelli et al., 2025). In addition, TB-PJI may coexist with common bacterial pathogens, so a positive routine bacterial culture does not necessarily exclude concomitant MTBC infection (Goh and Parvizi, 2022; Shi et al., 2024).

Metagenomic next-generation sequencing (mNGS) enables broad, culture-independent detection of microbial nucleic acids directly from clinical specimens (Street et al., 2017; Ivy et al., 2018; Liu and Ma, 2024; Zhao et al., 2024). In PJI and other complex infections, mNGS has generally shown a higher pathogen-detection rate than conventional culture, particularly in culture-negative or polymicrobial settings (Wang et al., 2020; Liu and Ma, 2024; Huang et al., 2025; Olearo et al., 2025). However, pathogen-detection yield should not be equated with complete diagnostic accuracy when the study population is selected by final etiological classification or when mNGS contributes to that classification.

Previous institutional studies reported a small number of MTBC-positive cases within broader polymicrobial PJI or culture-negative osteoarticular infection cohorts (Mei et al., 2023; Ding et al., 2024), and recent dedicated reports have evaluated tuberculous orthopedic implant-associated infection and TB-PJI (Auñon et al., 2024; Wang et al., 2024). Nevertheless, direct within-cohort comparison of TB-PJI with CB-PJI integrating pathogen-detection yield, synovial inflammatory characteristics, and comparative clinical phenotyping remains limited. Accordingly, the present study aimed to (1) compare the paired pathogen-detection yield of mNGS and conventional culture; (2) characterize the clinical, laboratory, and pathogen-spectrum features of TB-PJI, including mixed infection; and (3) explore clinical associations with TB-PJI using parsimonious Firth regression. These analyses were intended to be hypothesis-generating rather than to establish a validated prediction model or diagnostic cutoff.

## 2 Materials and methods

### 2.1 Study design and population selection

This single-center retrospective cohort study was approved by the Institutional Review Board of our hospital (Approval No.: MTCA, ECFAH of FMU [2015]084-2). Patients who underwent diagnosis and management for PJI at our institution between January 2016 and June 2024 were retrospectively screened for eligibility.

#### 2.1.1 Inclusion and exclusion criteria

The inclusion criteria were: (1) fulfillment of the 2018 International Consensus Meeting (ICM) diagnostic criteria for PJI; (2) concurrent conventional microbiological culture and mNGS testing using specimens collected during the same diagnostic or surgical episode; and (3) a definitive final etiological diagnosis of either common bacterial periprosthetic joint infection (CB-PJI) or tuberculous periprosthetic joint infection (TB-PJI) (Parvizi et al., 2018).

The exclusion criteria were defined as:

Presence of a concurrent malignant neoplasm; PJI cases with no identifiable pathogen (culture-negative and mNGS-negative with unconfirmed etiology); Infections caused by atypical or rare pathogens falling outside the classifications of CB-PJI or TB-PJI (e.g., fungal infections); A postoperative follow-up period of less than 2 years; or Incomplete clinical registry data or documented specimen contamination.

Ultimately, 197 patients were enrolled (178 CB-PJI and 19 TB-PJI), all fulfilling the 2018 ICM criteria for PJI. TB-PJI was retrospectively adjudicated using the totality of available diagnostic evidence. Because mNGS contributed to etiological classification in some cases, incorporation bias cannot be excluded, and results are therefore presented as pathogen-detection yield rather than diagnostic accuracy. Three TB-PJI patients (15.8%) had appeared in previous institutional publications addressing different research questions (Mei et al., 2023; Ding et al., 2024). The participant-selection process is summarized in **Supplementary Figure S2**.

To characterize heterogeneity in etiological certainty, TB-PJI cases were descriptively grouped according to the strength of TB-specific evidence: independent MTBC-specific confirmation by mycobacterial culture, TB-PCR, or Xpert MTB/RIF; mNGS-detected MTBC with supportive evidence but without independent MTBC-specific confirmation; or limited etiological evidence. This grouping was descriptive and was not intended as a validated diagnostic classification. A sensitivity analysis excluding the two cases with limited etiological evidence (Cases 16 and 19) was performed.

### 2.2 Specimen collection and processing

Specimen collection and processing followed our previously established protocol (Mei et al., 2023). Clinical specimens included synovial fluid, periprosthetic tissue, and sonication fluid. Culture and mNGS samples were obtained during the same diagnostic or surgical episode. Synovial fluid was divided into aliquots for parallel testing, whereas separate periprosthetic tissue samples from the same anatomical site were used for culture and mNGS. Retrieved prostheses were sonicated at 40 kHz for 5 minutes, and the resulting sonication fluid was used for microbiological and molecular analyses. Specimens were transported to the laboratory within 30 minutes and stored at −80 °C when archived.

### 2.3 Conventional microbiological culture

#### 2.3.1 Routine bacterial culture

Synovial fluid specimens were directly inoculated into BACTEC Plus/F aerobic and anaerobic blood culture bottles (Becton, Dickinson and Company, Franklin Lakes, NJ, USA) at the bedside or immediately upon arrival at the laboratory, and continuously monitored using the BACTEC FX automated blood culture system (Lyu et al., 2024). Periprosthetic tissue specimens were homogenized using a high-speed tissue homogenizer (JXFSTPRP-24; Jingxin, Shanghai, China) at 40 Hz for 60–90 seconds to maximize bacterial release from the tissue matrix (Wang et al., 2024). The resulting tissue homogenates were inoculated onto Columbia blood agar plates (Hycon Biological, Qingdao, China) and incubated at 35 °C under both aerobic and anaerobic conditions for up to 14 days to capture slow-growing organisms (Osmon et al., 2013).

To disrupt mature biofilms, intraoperative explanted prosthetic components were sonicated in 400 mL of sterile saline (40 kHz, 5 minutes), and a 10-mL aliquot of the sonication fluid was inoculated into the aforementioned BACTEC blood culture bottles. All positive cultures were isolated and identified to the species level using matrix-assisted laser desorption/ionization time-of-flight mass spectrometry (MALDI-TOF MS; Bruker Daltonik GmbH, Bremen, Germany). Antimicrobial susceptibility testing (AST) was performed in accordance with the Clinical and Laboratory Standards Institute (CLSI) guidelines.

#### 2.3.2 Mycobacterial culture

Mycobacterial culture was performed following the methodology described in our center’s previous study (Lyu et al., 2024). Clinical specimens were transferred to a designated, Biosafety Level 3 (BSL-3) tuberculosis laboratory at Fuzhou Pulmonary Hospital (Fuzhou, China) for specialized processing. After digestion and decontamination using the standard N-acetyl-L-cysteine–sodium hydroxide (NALC-NaOH) method, the treated sediment was simultaneously inoculated into BACTEC MGIT 960 liquid medium (Becton, Dickinson and Company, USA) and Lowenstein–Jensen solid medium. The media were incubated for up to 42 and 56 days, respectively, with a maximum extended observation period of 12 weeks before reporting a sample as negative for mycobacteria. Positive cultures were confirmed as Mycobacterium tuberculosis complex (MTBC) via acid-fast staining, MTBC-specific nucleic acid amplification testing (TB-PCR), and DNA microarray-based species identification. Drug susceptibility profiles were determined by integrating phenotypic AST results with genotypic drug-resistance mutations detected by the DNA microarray.

### 2.4 mNGS testing and data interpretation

Laboratory workflows and bioinformatic pipelines followed the established diagnostic protocols published by our center (Mei et al., 2023).

Nucleic Acid Extraction: A 500-μL aliquot of liquid specimen (synovial fluid or sonication fluid) or tissue homogenate supernatant was processed for DNA extraction using the TIANamp Micro DNA Kit (DP316; Tiangen Biotech, Beijing, China). DNA-based testing included a contemporaneous batch negative/background control, a positive control, and extraction quality control. The negative/background control consisted of HeLa-cell genomic DNA without intentionally added microbial templates, whereas the positive control contained Klebsiella pneumoniae together with HeLa cells.

Library Construction and Sequencing: Extracted DNA was fragmented, end-repaired, A-tailed, adapter-ligated, and PCR-amplified to construct sequencing libraries. High-throughput sequencing was performed using the validated BGI/MGI platform in use by the diagnostic laboratory during the corresponding testing period, generally with 50-bp single-end sequencing and a target depth of at least 20 million reads per sample. Because the cohort spanned 2016-2024, platform, reagent-lot, database, and analytical updates may have occurred over time; archived read counts were therefore interpreted within the contemporaneous laboratory workflow and were not considered directly quantitative measures of pathogen burden across patients.

Bioinformatics Analysis: Raw sequencing data were filtered to remove low-quality reads, low-complexity reads, adapter sequences, and reads shorter than 35 bp. High-quality reads were mapped against the human reference genome using the Burrows-Wheeler Aligner (BWA) software to filter out host sequences. The remaining non-host reads were aligned to the National Center for Biotechnology Information (NCBI) public microbial database for taxonomic annotation and classification.

The interpretation criteria for mNGS reports were based on our center’s established framework together with contemporaneous batch-control assessment and clinical relevance (Mei et al., 2023):

General low-abundance microbial signals were assessed relative to the contemporaneous negative/background control. As a general laboratory rule, when the stringently mapped read number (SMRN) was ≤50, the sample signal was generally required to be at least fivefold higher than the corresponding negative-control read count; when SMRN was >50, a threefold threshold was used. Known environmental and reagent-associated background organisms were filtered using batch-control information.

For common pathogens, species-specific mapped read number, genomic coverage, negative-control background, and clinical plausibility were considered together when determining whether a microorganism was reported as potentially pathogenic.

For MTBC, a pathogen-specific low-read approach was used because of the typically low organism burden. A finding of ≥1 stringently mapped MTBC read could be reported only after contemporaneous batch negative-control assessment and clinical correlation; a low read count alone was not regarded as automatic confirmation of TB-PJI. In the lowest-abundance MTBC-positive case, one stringently mapped MTBC read was detected, whereas the corresponding batch negative control contained zero MTBC reads. If MTBC reads were also present in the negative control and contamination could not be reliably excluded, the signal was not treated as a clinically meaningful MTBC finding and repeat testing could be considered.

Raw sequencing reads were generated independently of clinical information; clinical information was used only for interpretation of pathogenic relevance and final reporting. Reported mNGS findings were reviewed for clinical concordance by a multidisciplinary team including orthopedic infection specialists, an infectious disease physician, and a clinical microbiologist.

### 2.5 Data collection

The following clinical, laboratory, and surgical variables were retrospectively extracted from the institutional electronic medical record system:

Demographic characteristics: age, sex, body mass index (BMI), and Charlson Comorbidity Index (CCI). The original Charlson Comorbidity Index (CCI), without age adjustment, was calculated from comorbidities documented at the index hospitalization.

Clinical and surgical features: anatomical type of the infected joint, history of prior joint arthroplasty or invasive joint procedures, presence of sinus tract formation, and documentation of polymicrobial or mixed co-infections;

Laboratory indices: peripheral white blood cell (WBC) count, C-reactive protein (CRP), erythrocyte sedimentation rate (ESR), synovial fluid white blood cell (SF WBC) count, and synovial fluid polymorphonuclear neutrophil percentage (SF PMN%);

Therapeutic interventions and outcomes: surgical modalities, antimicrobial or anti-tuberculous treatment and treatment duration, total length of hospital stay, drug-related complications, and infection resolution or recurrence during follow-up. A patient was classified as having a drug-related complication if at least one treatment-associated adverse event was documented in the medical record. Surgical complications were not included in this endpoint, and no patient had more than one recorded drug-related complication. Detailed historical drug-susceptibility and regimen-modification information was recorded only when reliably retrievable from the source medical records.

For outcome analyses, follow-up was calculated from the date of the index surgical treatment. Persistent infection was defined as failure to achieve infection control after treatment, whereas infection recurrence was defined as reappearance of infection after a documented interval of clinical improvement. Patients without recurrence were censored at 24 months.

### 2.6 Statistical analysis

Statistical analyses were performed using SPSS version 26.0 (IBM Corp., Armonk, NY, USA) and R version 4.2.1 (R Foundation for Statistical Computing, Vienna, Austria).

Continuous variables were presented as mean ± SD or median (IQR) and compared using the independent-samples t-test or Mann–Whitney U test, as appropriate. Categorical variables were expressed as n (%) and compared using the χ^2^ test or Fisher’s exact test. Paired pathogen-detection rates between mNGS and conventional culture were compared using the two-sided McNemar test. Because mNGS contributed to TB-PJI adjudication in some cases, diagnostic accuracy measures were not estimated.

Given the limited number of TB-PJI events (n=19), regression analyses were considered exploratory and model complexity was deliberately restricted. The primary Firth penalized logistic regression model included three clinically interpretable and nonredundant variables: CCI (per 1-point increase), representing comorbidity burden; SF PMN% (per 10% increase), representing the local synovial inflammatory response; and BMI (per 5 kg/m^2^ increase). Variables closely related to microbiological phenotype or etiological classification, such as polymicrobial/mixed infection, were not included in the multivariable model. A secondary reduced Firth model including CCI and SF PMN% was also evaluated to assess directional consistency (**Supplementary Figure S1**). CCI data were complete for all patients. A small number of missing BMI and SF PMN% values were handled using multiple imputation, and all 197 patients were included in the Firth regression analysis. Adjusted odds ratios (aORs) with 95% CIs are reported. No diagnostic cutoff was derived. All tests were two-sided, with P<0.05 considered statistically significant.

## 3 Results

### 3.1 Baseline demographics and clinical characteristics

A total of 197 patients were included, comprising 178 patients with CB-PJI and 19 with TB-PJI (**Table 1**). Age did not differ significantly between the groups (66.08 ± 10.90 years vs. 70.32 ± 11.24 years, P = 0.110), nor did sex distribution (male: 43.8% vs. 36.8%, P = 0.559).

**Table 1:** Baseline demographics, clinical, and laboratory characteristics of CB-PJI vs. TB-PJI.

Characteristics	CB-PJI (n=178)	TB-PJI (n=19)	P-value
Age (years)	66.08 ± 10.90	70.32 ± 11.24	0.110
Gender (male/female)	78/100 (43.8%/56.2%)	7/12 (36.8%/63.2%)	0.559
BMI (kg/m^2^)	24.32 ± 3.23	22.53 ± 2.47	0.020*
Joint (hip/knee)	94/84 (52.8/47.2%)	13/6 (68.4/31.6%)	0.194
CCI	2.41 ± 1.33	4.26 ± 1.59	<0.001*
PIP (y/n)	113/65 (63.5/36.5%)	15/4 (78.9/21.1%)	0.179
Sinus tract (y/n)	46/132 (25.8/74.2%)	10/9 (52.6/47.4%)	0.014*
Polymicrobial/mixed infection (y/n)	28/150 (15.7/84.3%)	10/9 (52.6/47.4%)	<0.001*
Laboratory data					
WBC (×10^9^/L), median (IQR)	6.60 (5.23–8.68)	4.86 (3.80–8.16)	0.041
ESR (mm/h), median (IQR)	63.00 (38.00–89.00)	52.00 (28.25–85.25)	0.310
CRP (mg/L), median (IQR)	34.46 (12.10–65.80)	29.95 (10.70–66.35)	0.719
SF WBC (×10^6^/L), median (IQR)	11,895 (4,629.25–36,886.5)	82,240 (4,092.75–136,571.5)	0.055
SF PMN (%), median (IQR)	78.85 (67.75–89.93)	68.45 (34.90–83.33)	0.029

Data are presented as mean ± standard deviation, median (interquartile range), or n (%).

CB-PJI, common bacterial periprosthetic joint infection; TB-PJI, tuberculous periprosthetic joint infection; BMI, body mass index; CCI, Charlson Comorbidity Index; PIP, prior invasive procedure; WBC, white blood cell; ESR, erythrocyte sedimentation rate; CRP, C-reactive protein; SF WBC, synovial fluid white blood cell; SF PMN%, synovial fluid polymorphonuclear neutrophil percentage.

Independent-samples t-test or Mann-Whitney U test for continuous variables; χ^2^ test or Fisher’s exact test for categorical variables.

Statistically significant (*P<0.05).The asterisk (*) denotes statistical significance: P < 0.05.

Compared with CB-PJI, TB-PJI was associated with a lower mean BMI (22.53 ± 2.47 vs. 24.32 ± 3.23 kg/m2, P = 0.020) and a higher mean CCI score (4.26 ± 1.59 vs. 2.41 ± 1.33, P<0.001). Sinus tract formation (52.6% vs. 25.8%, P = 0.014) and polymicrobial/mixed infection (52.6% vs. 15.7%, P<0.001) were also more frequent in TB-PJI. Peripheral WBC count and SF PMN% were lower in TB-PJI and are reported as median (IQR): WBC, 4.86 (3.80-8.16) ×109/L versus 6.60 (5.23-8.68) ×109/L (P = 0.041); SF PMN%, 68.45% (34.90-83.33) versus 78.85% (67.75-89.93) (P = 0.029).

### 3.2 Comparison of pathogen-detection yield

CB-PJI Group: mNGS detected pathogens in 168/178 CB-PJI cases (94.38%), compared with 139/178 (78.09%) by conventional culture (P<0.001). Among the 39 culture-negative CB-PJI cases, mNGS identified a pathogen in 29 (74.4%), representing additional pathogen detection within this selected cohort.

TB-PJI Group (**Table 2**): MTBC was recovered by mycobacterial culture in 5/19 TB-PJI cases (26.3%) and detected by mNGS in 17/19 (89.5%; McNemar P<0.001). Five cases were positive by both methods, 12 were mycobacterial-culture-negative/mNGS-positive, none were mycobacterial-culture-positive/mNGS-negative, and two were negative by both methods. Thus, mNGS provided additional MTBC detection in 12/19 cases (63.2%) not recovered by mycobacterial culture. Patient-level mNGS read counts and complementary TB diagnostic evidence are summarized in **Supplementary Table S1**. Because mNGS contributed to etiological adjudication in some patients, these values are reported as pathogen-detection yield rather than sensitivity against an independent reference standard.

**Table 2:** Paired comparison of MTBC detection by mycobacterial culture versus mNGS in the TB-PJI subgroup (McNemar test).

MTBC detection measure	TB group (N = 19)	P (McNemar, two-sided)
MTBC culture positive, n/N (%)	5/19 (26.3%)	--
mNGS MTBC positive, n/N (%)	17/19 (89.5%)	< 0.001
MTBC culture−/mNGS+ (additional detection^a^), n/N (%)	12/19 (63.2%)	--
mNGS−/MTBC culture+ (discordant^b^), n/N (%)	0/19 (0.0%)	--
Overall agreement^c^, n/N (%)	7/19 (36.8%)	--

Data are presented as n/N (%).

MTBC, Mycobacterium tuberculosis complex; mNGS, metagenomic next-generation sequencing.

^a^Additional detection: proportion of TB-PJI cases in which MTBC was detected by mNGS but not recovered by mycobacterial culture.

^b^Discordant cases: proportion of TB-PJI cases positive by mycobacterial culture but negative by mNGS.

^c^Overall agreement: proportion of cases with concordant positive or concordant negative MTBC results.

P<0.05 was considered statistically significant.The asterisk (*) denotes statistical significance: P < 0.05.

Among the 19 TB-PJI cases, eight had independent MTBC-specific confirmation, nine had mNGS-detected MTBC together with supportive evidence but no independent MTBC-specific confirmation, and two cases (Cases 16 and 19) had more limited etiological evidence. In a sensitivity analysis excluding Cases 16 and 19, mNGS detected MTBC in 16/17 cases (94.1%), compared with 5/17 (29.4%) by mycobacterial culture. Eleven cases were mNGS-positive/mycobacterial-culture-negative, whereas none were mNGS-negative/mycobacterial-culture-positive (exact McNemar P<0.001).

### 3.3 Pathogen spectrum analysis

In the CB-PJI group, the overall pathogen spectrum was dominated by Staphylococcus species, with Staphylococcus epidermidis (22.5%) and Staphylococcus aureus (21.1%) the most frequent pathogen occurrences (**Figure 1**). Other organisms included Klebsiella pneumoniae, Staphylococcus haemolyticus, Escherichia coli, and additional bacterial species.

**Figure 1:**
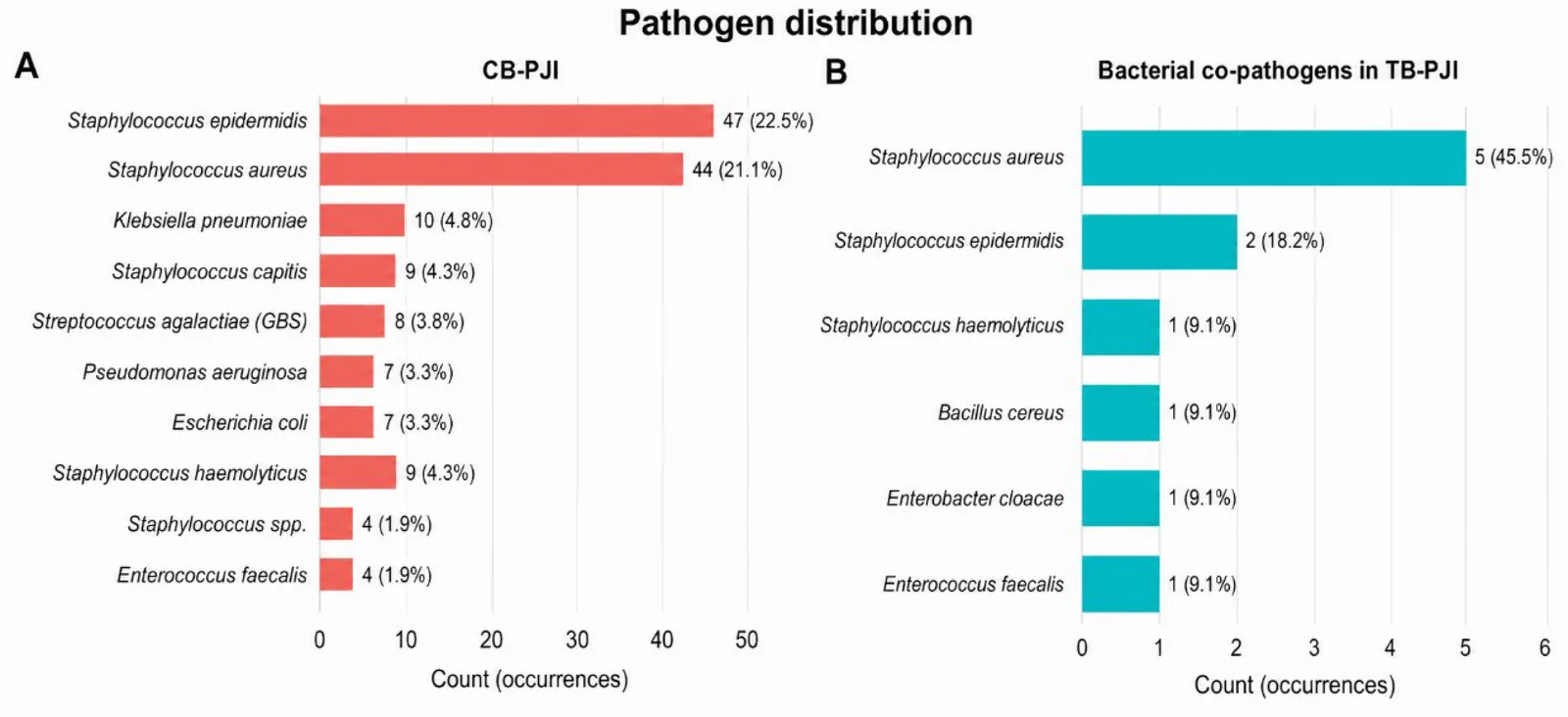
Pathogen distribution in CB-PJI **(A)** and bacterial co-pathogen distribution in TB-PJI with mixed infection **(B)**. In **(A)**, bars represent pathogen-occurrence counts in CB-PJI, and percentages are calculated using the total number of pathogen occurrences in the CB-PJI group (n=209). In **(B)**, bars represent non-MTBC bacterial co-pathogen occurrences among TB-PJI cases with mixed infection, and percentages are calculated using the total number of non-MTBC bacterial co-pathogen occurrences in TB-PJI (n=11).

In the TB-PJI group, polymicrobial/mixed infection was identified in 10 of 19 patients (52.6%). These mixed infections comprised 11 non-MTBC bacterial co-pathogen occurrences. Staphylococcus aureus was the most frequent co-pathogen (5/11), followed by Staphylococcus epidermidis (2/11).

### 3.4 Clinical outcomes and complications

Patients with TB-PJI had a longer hospital stay [median 30 vs. 18 days, P<0.001] and a longer duration of antimicrobial/anti-tuberculous therapy [median 408 vs. 94 days, P<0.001] (**Table 3**). Drug-related complications were more frequent in the TB-PJI group than in the CB-PJI group (42.1% vs. 11.8%, P = 0.002). Among the eight affected TB-PJI patients, the recorded complications included drug-induced liver injury (n=3), drug-related fever (n=2), gastrointestinal adverse events (n=2), and rash (n=1). The 2-year infection recurrence rate was 15.8% (3/19) in the TB-PJI group and 7.9% (14/178) in the CB-PJI group; the difference was not statistically significant (Fisher exact P = 0.216). The three TB-PJI recurrence events occurred at 1, 2, and 24 months after the index surgical treatment, respectively. All three events occurred after a documented interval of infection control rather than representing persistent infection.

**Table 3:** Comparison of treatment strategies and clinical outcomes between the two groups.

Variables	CB-PJI (n=178)	TB-PJI (n=19)	P-value
Follow-up period (months)	45.14 ± 23.20	47.63 ± 24.90	0.659
Treatment, n (%)			0.070
DAIR	35 (19.7%)	6 (31.6%)	
One-stage revision	52 (29.2%)	1 (5.3%)	
Staged revision^c^	91 (51.1%)	12 (63.2%)	
Length of hospital stay, days (IQR)	18 (15–22)	30 (25–37)	<0.001*
Duration of antimicrobial/anti-tuberculous therapy, days (IQR)	94 (60–110)	408 (306–435)	<0.001*
Drug-related complications (y/n)	21/157 (11.8%/88.2%)	8/11 (42.1%/57.9%)	0.002*
Infection recurrence (y/n)	14/164 (7.9%/92.1%)	3/16 (15.8%/84.2%)	0.216

Data are presented as mean ± standard deviation, median (interquartile range), or n (%).

CB-PJI, common bacterial periprosthetic joint infection; TB-PJI, tuberculous periprosthetic joint infection; DAIR, debridement, antibiotics, and implant retention; IQR, interquartile range.

Mann-Whitney U test for continuous variables; χ^2^ test or Fisher’s exact test for categorical variables.

Statistically significant (*P<0.05).

^c^For group-level statistical analysis, the TB-PJI staged-revision group included 11 two-stage revision cases and one 1.5-stage revision case.The asterisk (*) denotes statistical significance: P < 0.05.

### 3.5 Exploratory Firth regression analysis

In the primary exploratory Firth model, each 1-point increase in CCI was associated with higher odds of TB-PJI (aOR=2.39, 95% CI: 1.51–4.10, P<0.001), whereas each 10% increase in SF PMN% was associated with lower odds of TB-PJI (aOR=0.722, 95% CI: 0.564–0.917, P = 0.009) (**Figure 2**). BMI was not statistically significant (aOR=0.569, 95% CI: 0.186–1.576, P = 0.281) (**Figure 2**). The secondary reduced model excluding BMI yielded similar effect estimates and preserved the direction of association for both CCI and SF PMN% (**Supplementary Figure S1**).

**Figure 2:**
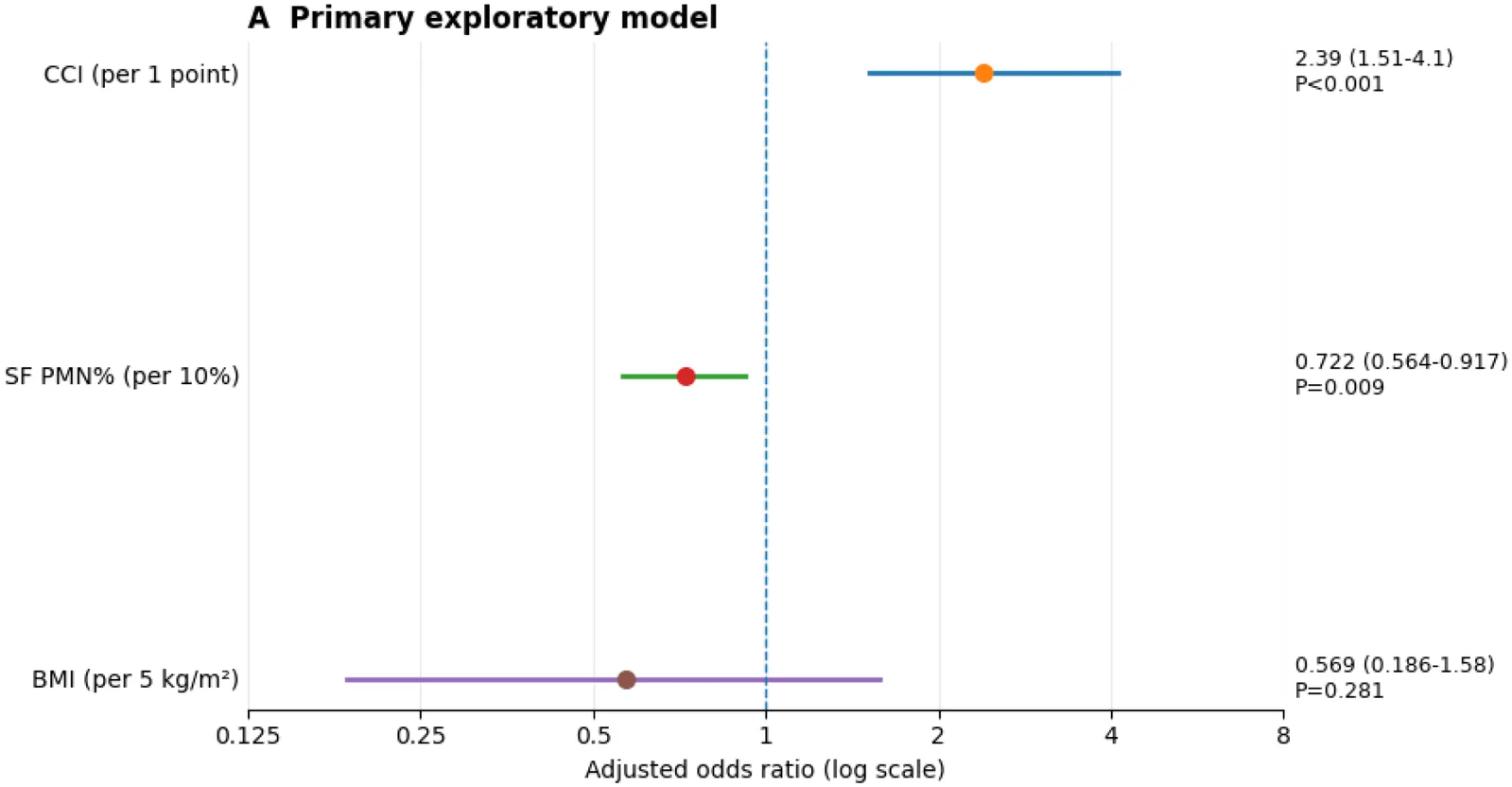
Forest plot of the primary exploratory Firth logistic regression model for TB-PJI. The model included CCI per 1-point increase, SF PMN% per 10% increase, and BMI per 5 kg/m^2^ increase. aOR, adjusted odds ratio; CI, confidence interval; CCI, Charlson Comorbidity Index; BMI, body mass index; SF PMN%, synovial fluid polymorphonuclear neutrophil percentage.

### 3.6 Survival analysis

Kaplan-Meier analysis showed no statistically significant difference in 2-year infection control between the CB-PJI and TB-PJI groups (log-rank P = 0.218) (**Figure 3**). This finding should not be interpreted as demonstrating equivalence between the groups.

**Figure 3:**
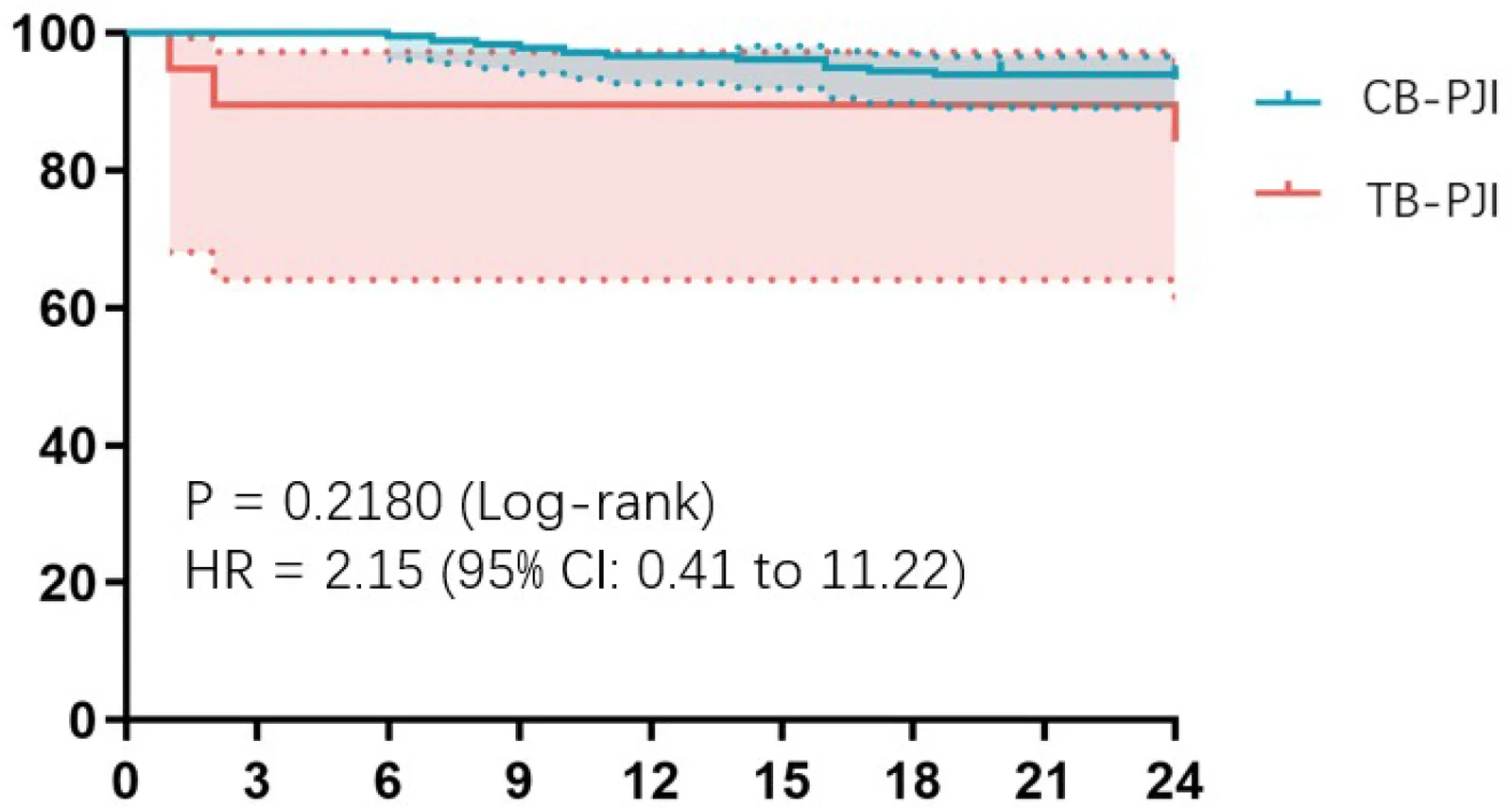
Kaplan-Meier curves for 2-year infection control in CB-PJI and TB-PJI. No statistically significant difference was observed between groups (log-rank P = 0.218).

## 4 Discussion

This study compared pathogen-detection yield and clinical characteristics between TB-PJI and CB-PJI in a selected retrospective cohort. The principal findings were that mNGS detected MTBC in substantially more TB-PJI cases than mycobacterial culture; TB-PJI was characterized by a higher comorbidity burden, lower SF PMN%, and more frequent mixed infection; continuous CCI and SF PMN% showed exploratory associations with TB-PJI in Firth regression; and TB-PJI required longer treatment and was associated with more drug-related complications, while 2-year infection recurrence did not differ significantly between groups.

mNGS detected MTBC in 17/19 TB-PJI cases, whereas mycobacterial culture recovered MTBC in 5/19, with 12 additional mNGS-positive/mycobacterial-culture-negative cases. This supports a higher pathogen-detection yield for mNGS in this selected cohort and is consistent with reports describing the utility of mNGS in culture-negative PJI and tuberculous orthopedic implant-associated infection (Wang et al., 2020; Shi et al., 2024; Wang et al., 2024; Huang et al., 2025; Olearo et al., 2025). However, these proportions should not be interpreted as sensitivity estimates against an independent gold standard. The cohort excluded unresolved etiologies and other selected infection groups, and mNGS contributed to final TB-PJI adjudication in some patients; therefore, specificity, predictive values, and complete diagnostic accuracy cannot be established from this design.

Low-abundance MTBC findings also require cautious interpretation. The one-read MTBC-positive case had zero MTBC reads in its contemporaneous batch negative control and had additional supportive clinical evidence, as detailed in **Supplementary Table S1**. More generally, read count alone was not treated as automatic confirmation of TB-PJI; batch-control assessment and the totality of microbiological, molecular, histopathological, and clinical evidence were considered together. mNGS should therefore be regarded as an adjunct to mycobacterial culture, TB-PCR/Xpert, and histopathology rather than a stand-alone diagnostic test.

CCI modeled continuously per 1-point increase was associated with higher odds of TB-PJI, whereas SF PMN% modeled per 10% increase was associated with lower odds; BMI was not statistically significant. The secondary reduced model excluding BMI yielded directionally consistent associations (**Supplementary Figure S1**). Given the limited number of TB-PJI events (n=19), these findings should be interpreted as exploratory associations requiring external validation. No diagnostic cutoff was derived or proposed. The biological mechanisms underlying these clinical differences were not directly tested in the present cohort.

Mixed infection was present in 10/19 TB-PJI patients (52.6%), comprising 11 non-MTBC bacterial co-pathogen occurrences. Staphylococcus aureus was the most frequent co-pathogen (5/11), followed by Staphylococcus epidermidis (2/11). Clinically, recovery of a common bacterial pathogen does not necessarily exclude concomitant TB when the clinical course or complementary findings remain suspicious. The present data do not establish a specific ecological or biofilm interaction between MTBC and bacterial co-pathogens.

TB-PJI was associated with longer hospitalization, longer antimicrobial/anti-tuberculous treatment, and more drug-related complications. Nevertheless, the difference in 2-year infection recurrence was not statistically significant, and Kaplan-Meier analysis likewise showed no statistically significant difference in infection control. These findings do not demonstrate equivalence between TB-PJI and CB-PJI and do not establish that mNGS reduced diagnostic delay, antimicrobial exposure, recurrence, or improved long-term outcomes (Osmon et al., 2013; Lo et al., 2021).

Several limitations should be considered. First, this was a single-center retrospective study with only 19 TB-PJI cases; Firth penalization reduces small-sample bias but does not overcome the uncertainty associated with the limited event count (Heinze and Schemper, 2002). Second, participant selection based on final etiological classification excluded unresolved cases lacking independent etiological confirmation and selected other etiological groups, preventing estimation of specificity, PPV, NPV, or complete diagnostic accuracy. Third, incorporation bias cannot be excluded because mNGS contributed to TB-PJI adjudication in some patients, and not all mNGS-positive TB-PJI cases had independent MTBC-specific microbiological or molecular confirmation. Fourth, the long study period encompassed platform, reagent-lot, database, and workflow updates, so archived mNGS read counts are not directly quantitative across patients. Fifth, detailed historical drug-susceptibility and regimen-modification data were not consistently retrievable for all patients and were not inferred when unavailable. Finally, formal cost-effectiveness analysis was not performed. Larger prospective multicenter cohorts are required to validate the observed clinical associations and to define standardized implementation strategies for mNGS in TB-PJI.

## 5 Conclusion

In this selected retrospective cohort, mNGS showed a higher pathogen-detection yield than conventional culture, particularly in TB-PJI, and identified MTBC in cases not recovered by mycobacterial culture. Higher CCI and lower SF PMN% were exploratory clinical associations with TB-PJI; these associations require external validation and were not used to derive diagnostic thresholds. mNGS is best considered an adjunct to mycobacterial culture, TB-specific molecular testing, and histopathology, and the present findings require external validation in larger prospective cohorts.

## Data Availability

The original contributions presented in the study are included in the article/**Supplementary Material**. Further inquiries can be directed to the corresponding authors.

## Supplementary material

Supplementary Figure 1 Forest plot of the secondary reduced Firth logistic regression model for TB-PJI. The reduced model included CCI per 1-point increase and SF PMN% per 10% increase. aOR, adjusted odds ratio; CI, confidence interval; CCI, Charlson Comorbidity Index; SF PMN%, synovial fluid polymorphonuclear neutrophil percentage.

Supplementary Figure 2 Flow diagram of study participant selection. A total of 304 patients were assessed for eligibility. After exclusion of patients according to the predefined criteria, 197 patients were included in the final selected cohort, including 178 with common bacterial periprosthetic joint infection (CB-PJI) and 19 with tuberculous periprosthetic joint infection (TB-PJI).

Supplementary Table S1 Patient-Level mNGS Results, Strength of Etiological Evidence, Additional Diagnostic Findings, and Clinical Outcomes of 19 TB-PJI Cases.
