# Supplementary material for: Metagenomic next-generation sequencing in tuberculous periprosthetic joint infection: pathogen-detection yield and clinical characteristics compared with common bacterial PJI
Source: Front Cell Infect Microbiol. 2026 Oct 7;16:1935713. doi: 10.3389/fcimb.2026.1935713 (PMC13683129; doi:10.3389/fcimb.2026.1935713)
Supplement: Supplementary file 1 [file SupplementaryFile1.docx]

**Supplementary Table S1. Patient-Level mNGS Results, Strength of Etiological Evidence, Additional Diagnostic Findings, and Clinical Outcomes of 19 TB-PJI Cases**

| **Case** | **mNGS result** | **Additional diagnostic findings** | **Treatment / outcome** | **Strength of etiological evidence** |
| --- | --- | --- | --- | --- |
| **1** | Periprosthetic tissue; MTBC 3,556,351 reads | Chronic granulomatous inflammation; conventional bacterial culture: *Staphylococcus aureus* (+); MTBC culture (−) | Two-stage revision + anti-tuberculous therapy; no recurrence at 2 years | mNGS + supportive evidence |
| **2** | Synovial fluid; MTBC 1,627 reads | Chronic granulomatous inflammation with multinucleated giant cells; T-SPOT.TB (−); MTBC culture (−) | Recurrence at 1 month after the index surgical treatment; infection controlled following subsequent two-stage revision + anti-tuberculous therapy | mNGS + supportive evidence |

| **3** | Synovial fluid; MTBC 623 reads | Chronic granulomatous inflammation; conventional bacterial culture: *Staphylococcus haemolyticus* (+); MTBC culture (−) | Two-stage revision + anti-tuberculous therapy; no recurrence at 2 years | mNGS + supportive evidence |
| --- | --- | --- | --- | --- |
| **4** | Synovial fluid; MTBC 73 reads | MTBC culture (+); conventional bacterial culture: *Staphylococcus aureus* (+) | Two-stage revision + anti-tuberculous therapy; no recurrence at 2 years | Independent confirmation |
| **5** | Periprosthetic tissue; MTBC 57 reads | Chronic granulomatous inflammation; T-SPOT.TB (+); conventional bacterial culture: *Staphylococcus epidermidis* (+); MTBC culture (−) | One-stage revision + anti-tuberculous therapy; no recurrence at 2 years | mNGS + supportive evidence |
| **6** | Synovial fluid; MTBC 50 reads | Chronic granulomatous inflammation; T-SPOT.TB (+); MTBC culture (−) | Two-stage revision + anti-tuberculous therapy; no recurrence at 2 years | mNGS + supportive evidence |
| **7** | Periprosthetic tissue; MTBC 46 reads | MTBC culture (+) | Two-stage revision + anti-tuberculous therapy; no recurrence at 2 years | Independent confirmation |
| **8** | Synovial fluid; MTBC 31 reads | MTBC culture (+); chronic granulomatous inflammation; conventional bacterial culture: *Bacillus cereus* (+) | Two-stage revision + anti-tuberculous therapy; no recurrence at 2 years | Independent confirmation |
| **9** | Periprosthetic tissue; MTBC 22 reads | MTBC culture (+) | DAIR + anti-tuberculous therapy; no recurrence at 2 years | Independent confirmation |
| **10** | Sonication fluid; MTBC 18 reads | MTBC culture (+); conventional bacterial culture: *Staphylococcus aureus* (+) | Two-stage revision + anti-tuberculous therapy; recurrence at 2 months after the index surgical treatment | Independent confirmation |

| **11** | Synovial fluid; MTBC 4,355 reads | TB-PCR (+); conventional bacterial culture: *Staphylococcus aureus* (+); MTBC culture (−) | DAIR + anti-tuberculous therapy; no recurrence at 2 years | Independent confirmation |
| --- | --- | --- | --- | --- |
| **12** | Synovial fluid; MTBC 12 reads | Chronic granulomatous inflammation; MTBC culture (−); history of pulmonary tuberculosis | Two-stage revision + anti-tuberculous therapy; no recurrence at 2 years | mNGS + supportive evidence |
| **13** | Synovial fluid; MTBC 521 reads | TB-PCR (+); MTBC culture (−) | DAIR + anti-tuberculous therapy; no recurrence at 2 years | Independent confirmation |
| **14** | Periprosthetic tissue; MTBC 2 reads | Chronic granulomatous inflammation; conventional bacterial culture: *Enterobacter cloacae* (+); MTBC culture (−) | Two-stage revision + anti-tuberculous therapy; no recurrence at 2 years | mNGS + supportive evidence |
| **15** | Periprosthetic tissue; MTBC 2 reads | Chronic granulomatous inflammation; MTBC culture (−) | Two-stage revision + anti-tuberculous therapy; recurrence at 24 months after the index surgical treatment | mNGS + supportive evidence |
| **16** | Synovial fluid; MTBC 3,589 reads | Conventional bacterial culture: *Staphylococcus aureus* (+) and *Staphylococcus epidermidis* (+); MTBC culture (−); no additional independent TB-specific confirmation available | DAIR + anti-tuberculous therapy; no recurrence at 2 years | Limited etiological evidence |
| **17** | Periprosthetic tissue; MTBC 1 readᵃ | Chronic granulomatous inflammation with multinucleated giant cells; T-SPOT.TB (+); conventional bacterial culture: *Enterococcus faecalis* (+); MTBC culture (−) | 1.5-stage revisionᵇ + anti-tuberculous therapy; no recurrence at 2 years | mNGS + supportive evidence |
| **18** | mNGS negative | Xpert MTB/RIF (+); MTBC culture (−) | DAIR + anti-tuberculous therapy; no recurrence at 2 years | Independent confirmation |
| **19** | mNGS negative | Chronic granulomatous inflammation; acid-fast staining (+); MTBC culture (−) | DAIR + anti-tuberculous therapy; no recurrence at 2 years | Limited etiological evidence |

**Abbreviations:** MTBC, *Mycobacterium tuberculosis* complex; mNGS, metagenomic next-generation sequencing; TB-PCR, tuberculosis-specific polymerase chain reaction; DAIR, debridement, antibiotics, and implant retention; T-SPOT.TB, T-cell spot test for tuberculosis.

**Table notes:**

Cases are numbered according to the cohort entry timeline (diagnostic/surgical episode) rather than by sequencing read count order.

ᵃ In the lowest-abundance MTBC-positive case (Case 17), one stringently mapped MTBC read was detected, whereas no MTBC read was detected in the corresponding batch negative control.

ᵇ For group-level statistical analysis, the single 1.5-stage revision case was categorized within the staged-revision group.

Negative results indicate that the corresponding diagnostic test was actually performed and yielded a negative result. Tests not listed were either not performed or were not retrievable from the available medical records.

mNGS read counts represent MTBC reads recorded in the archived sequencing reports and should not be interpreted as directly quantitative measures of pathogen burden across patients, because sequencing depth, platform characteristics, reagent batches, database versions, and analytical workflows may have varied during the study period.

Additional diagnostic findings were interpreted according to their diagnostic nature. MTBC culture, TB-PCR, and Xpert MTB/RIF were considered microbiological or molecular evidence supporting active TB infection. Histopathological findings and acid-fast staining were considered supportive findings, whereas T-SPOT.TB positivity and a previous history of tuberculosis were not regarded as independent microbiological confirmation of active TB-PJI.

Strength-of-evidence categories were descriptive and were not intended as a validated diagnostic classification. "Independent confirmation" denotes MTBC-specific confirmation by mycobacterial culture, TB-PCR, or Xpert MTB/RIF. "mNGS + supportive evidence" denotes mNGS-detected MTBC together with supportive histopathological, immunological, or clinical evidence but without independent MTBC-specific confirmation. "Limited etiological evidence" denotes cases with more limited TB-specific etiological support.
