# Supplementary material for: Metagenomic next-generation sequencing in tuberculous periprosthetic joint infection: pathogen-detection yield and clinical characteristics compared with common bacterial PJI
Source: Front Cell Infect Microbiol. 2026 Oct 7;16:1935713. doi: 10.3389/fcimb.2026.1935713 (PMC13683129; doi:10.3389/fcimb.2026.1935713)
Supplement: Supplementary file 2 [file SupplementaryFile2.docx]

**Supplementary Figure S1.** Forest plot of the secondary reduced Firth logistic regression model for TB-PJI. The reduced model included CCI per 1-point increase and SF PMN% per 10% increase. aOR, adjusted odds ratio; CI, confidence interval; CCI, Charlson Comorbidity Index; SF PMN%, synovial fluid polymorphonuclear neutrophil percentage.
