# Supplementary material for: Metagenomic next-generation sequencing in tuberculous periprosthetic joint infection: pathogen-detection yield and clinical characteristics compared with common bacterial PJI
Source: Front Cell Infect Microbiol. 2026 Oct 7;16:1935713. doi: 10.3389/fcimb.2026.1935713 (PMC13683129; doi:10.3389/fcimb.2026.1935713)
Supplement: Supplementary file 3 [file SupplementaryFile3.docx]

**Supplementary Figure S2. Flow diagram of study participant selection.**
A total of 304 patients were assessed for eligibility. After exclusion of patients according to the predefined criteria, 197 patients were included in the final selected cohort, including 178 with common bacterial periprosthetic joint infection (CB-PJI) and 19 with tuberculous periprosthetic joint infection (TB-PJI).
